# Disrupting dorsolateral prefrontal cortex by rTMS reduces the P300 based marker of deception

**DOI:** 10.1002/brb3.656

**Published:** 2017-03-03

**Authors:** Inga Karton, Talis Bachmann

**Affiliations:** ^1^ Institute of PsychologyUniversity of TartuTartuEstonia; ^2^ Department of Penal LawSchool of LawUniversity of Tartu (Tallinn branch)TallinnEstonia; ^3^ Estonian National Defence CollegeTartuEstonia

**Keywords:** deception, dorsolateral prefrontal cortex, P300, repetitive transcranial magnetic stimulation

## Abstract

**Objective:**

Quite many studies have revealed certain brain‐process signatures indicative of subject's deceptive behavior. These signatures are neural correlates of deception. However, much less is known about whether these signatures can be modified by noninvasive brain stimulation techniques representing methods of causal intervention of brain processes and the corresponding behavior. Our purpose was to explore whether such methods have an effect on these signatures.

**Methods:**

It is well known that electroencephalographic event‐related potential component, P300, is sensitive to perception of critical items in a concealed information test, one of the central methods in deception studies. We examined whether the relative level of expression of P300 as a neural marker of deception can be manipulated by means of noninvasive neuromodulation. We used EEG/ERP recording combined with (i) neuronavigated repetitive transcranial magnetic stimulation (rTMS) and (ii) concealed information detection test. An opportunistically recruited volunteer group of normal adults formed our experimental group.

**Results:**

We show that offline rTMS to dorsolateral prefrontal cortex attenuated relative P300 amplitude in response to the critical items compared to the neutral items.

**Conclusion:**

Noninvasive prefrontal cortex excitability disruption by rTMS can be used to manipulate the sensitivity of ERP signatures of deception to critical items in a concealment‐based variant of lie detection test.

## 1 Introduction

Current brain imaging technology has allowed to demonstrate that neurobiological signatures of cognitive processes are different when involved in lying compared to when truthful behavior is the case (Ganis & Keenan, 2009; Ganis, Kosslyn, Stose, Thompson, & Yurgelun‐Todd, 2003; Jiang et al., 2013; Kozel et al., 2009; Langleben et al., 2005). This obvious fact makes it possible to develop objective methods of deception detection based on psychophysiology and brain imaging. In the various versions of the concealed information test (CIT), psychophysiological responses to critical items (termed also the relevant probe‐ or potentially incriminating items) are compared to the responses to neutral items (called also as irrelevant, contextually insignificant items). This procedure is applied when the subjects are trying to hide or deny that they have specific contextual knowledge of the critical items. If the critical items lead to enhanced responses compared to the responses to neutral items, possession of concealed information can be inferred.

Traditionally, CIT was used together with polygraph recordings, revealing enhanced respiratory and/or galvanic skin responses to critical items (e.g., Ben‐Shakhar & Elaad, 2003; Lykken, 1959, 1979). However, in a more modern tradition, the CIT is often combined with electroencephalography (EEG) in order to register deception‐related event‐related potentials (ERPs). For example, the ERP component, P300, is regarded as a relevant electrophysiological marker in the studies of deception (Ambach, Bursch, Stark, & Vaitl, 2010; Rosenfeld & Labkovsky, 2010; Verschuere, Ben‐Shakhar, & Meijer, 2011). If a deception‐related critical (probe) stimulus is presented, the P300 in response to this stimulus is enhanced compared to irrelevant stimuli (Ambach et al., 2010; Rosenfeld, Hu, Labkovsky, Meixner, & Winograd, 2013).

However, if viewed from the methodological point of view, the above mentioned electrophysiological measures of deception are correlational—brain imaging markers correlate with certain behavioral processes but causal effects cannot be definitely revealed. A somewhat different tradition of neurobiological research on deception combines brain imaging with noninvasive brain stimulation (reviews: Rogasch & Fitzgerald, 2013; Shafi, Westover, Fox, & Pascual‐Leone, 2012). This approach allows examining causal effects and therefore increases methodological rigor of the studies of brain mechanisms of deception (Gamer, Bauermann, Stoeter, & Vossel, 2007; Karton & Bachmann, 2011; Karton, Palu, Jõks, & Bachmann, 2014; Karton, Rinne, & Bachmann, 2014; Luber, Fisher, Appelbaum, Ploesser, & Lisanby, 2009; Mameli et al., 2010; Priori et al., 2008). Despite this potential, the studies examining the effects of brain stimulation on deception‐related P300 ERPs are difficult to find.

Several previous publications have reported that the dorsolateral prefrontal cortex (DLPFC) is involved in deceptive behavior (Christ, Van Essen, Watson, Brubaker, & McDermott, 2009; Ito et al., 2012; Mameli et al., 2010; Priori et al., 2008). In our earlier published studies (Karton & Bachmann, 2011; Karton, Palu, et al., 2014; Karton, Rinne, et al., 2014), we explored the causal effects of manipulation of DLPFC on deception‐related behavior. Repetitive 1‐Hz offline transcranial magnetic stimulation (rTMS) caused a change in the relative rate of untruthful responses when DLPFC was targeted. (*Offline* stimulation method means that TMS is applied before or after a subject performs the experimental task, but not during this task performance.) Because a change in the amplitude of ERP/P300 is the best known brain‐potential signature of deception in the concealed information detection test and because TMS has been shown to affect P300 in different contexts (Hansenne, Laloyaux, Mardaga, & Ansseau, 2004; Torii, Sato, Iwahashi, & Iramina, 2012), it would be important to know whether rTMS targeted at DLPFC has any effect on the extent of expression of P300 in the context of ERP‐based CIT. This is important both for theoretical analysis of the brain mechanisms involved in the subjects' behavior in the CIT‐like tasks and for practical purposes when manipulation with subjects' sensitivity to critical stimuli operationalized by deception‐related ERPs might be desirable. If rTMS can lead to higher sensitivity of ERPs to deception, the ERP‐based deception detection methods can be improved. Alternatively, if rTMS subdues the above mentioned ERP sensitivity, TMS‐based methods of building up resilience to CIT‐testing can be considered or the need to verify if the testees may have been rTMS‐stimulated must be acknowledged. The combination of CIT with EEG, supplemented by rTMS should be particularly well suited for pursuing these tasks. This combination allows a superior temporal resolution of the evoked neural processes in response to a crime‐related item, precise targeting of the brain areas likely involved in deception, and allows also studying causal effects in addition to the purely correlational brain imaging data.

To state the working hypotheses, we need also to consider specific information related to DLPFC‐targeted rTMS effects on deceptive behavior. On one hand it appears that especially right‐hemisphere rTMS targeted at DLPFC has stronger influence on deceptive behavior (although left DLPFC may be also involved, depending on task conditions) (Karton & Bachmann, 2011; Karton, Palu, et al., 2014; Karton, Rinne, et al., 2014). We therefore expect that rTMS of right DLPFC has a significant effect on P300 based markers of deception. On the other hand, clear disruptive rTMS effects on P300 have been found specifically with left‐hemisphere rTMS of DLPFC in the context different from deception detection (Torii et al., 2012). Thus, in order to have a clearer picture of the putative rTMS effects on P300 based deception markers in the CIT context, DLPFC of both hemispheres will be stimulated. We hypothesize that right but not left DLPFC rTMS will have an effect on the P300 difference between the conditions of neutral and critical stimulus presentation, whereas left DLPFC rTMS will change P300 parameters uniformly regardless of the stimulus type.

## 2 Method

### 2.1 Participants

All subjects who participated in our study were healthy and had normal or corrected to normal vision. They gave written informed consent before participation. The experiments were approved by the Research Ethics Committee of the University of Tartu and were conducted according to the principles set in the Declaration of Helsinki. Some of the subjects received monetary compensation for participation; others were awarded partial course credits.

Overall, there were 25 subjects (five males, 20 females) participating in the experiment. (As our sample was recruited opportunistically from the university environment with relatively more females present, the relative number of subjects agreeing to participate in the TMS experiment turned out to be unequal by gender. This can be considered as a limitation of this study, to be overcome in subsequent research.) Data of two male and five female subjects were excluded due to noisy EEG recordings and extensive blink artifacts. The age of the remaining 18 subjects ranged from 20 to 40 years (mean age 25.18 years, standard deviation (SD) 5.63 years). Subjects were randomly assigned into two stimulation groups: nine subjects (one male and eight females) received rTMS (repetitive transcranial magnetic stimulation) and sham stimulation targeted at left DLPFC and nine subjects (two males and seven females) received rTMS and sham stimulation targeted at right DLPFC.

### 2.2 Experimental procedures

Our experimental task was analogous to other variations of the Guilty Knowledge Test (GKT) and the Concealed Information Test (CIT). There were three categories of stimuli: critical (probe), neutral (unfamiliar in the experimental behavioral context), and familiar (i.e., familiar from the experimental behavioral context). ERP responses to seen stimuli belonging to each of these categories were to be compared. The experiment started with dramatizing a thieving episode (a “shoplifting” mock crime scenario). The purpose of the experiment as explained to the subjects was to discover “stealing” using EEG and a computer. The subject was motivated to hide the “crime”‐related knowledge. We used cards with words referring to five kinds of items “easy” to steal (e.g., chewing gum, candy, fruit, etc.). In each session, three words from five possible word alternatives written on cards were selected at random and put face up on a table next door. Subjects were instructed to enter that room and imagine that they are in the supermarket about to steal something. To do so, one of the three cards had to be taken. Subjects were instructed to write the name of the “stolen” item on the opposite side of the card and specify it more precisely, for example, by naming some favorite brand. Then this “critical” (probe) card had to be put into a folder (a “bag” for the stolen good) and the subjects brought it to the room where the TMS/ERP experiment begun. Subjects were told that the purpose of the experiment is to discover “stealing” using EEG recordings and they should hide their “crime”‐related knowledge.

**Figure 1:**
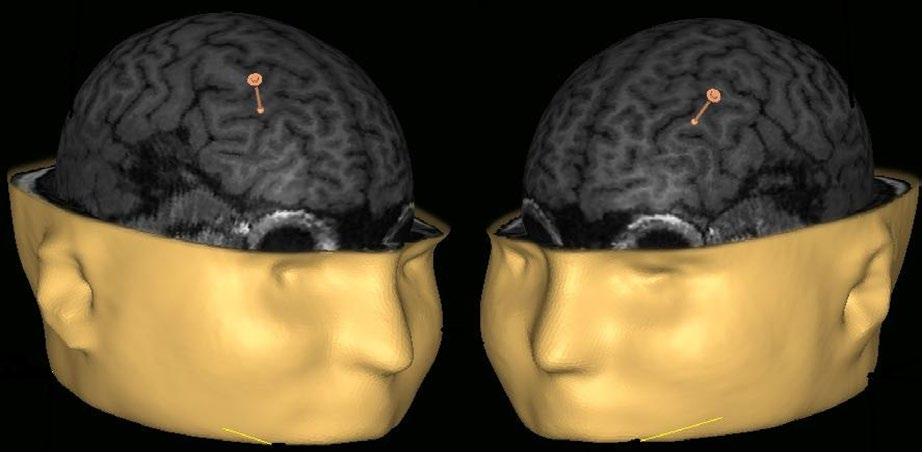
Illustration of localization of the rTMS target areas in the right and left DLPFC; rTMS stimulation was performed after the mock crime scenario (“shoplifting” enactment) and before the CIT task that was performed together with EEG recording

**Figure 2:**
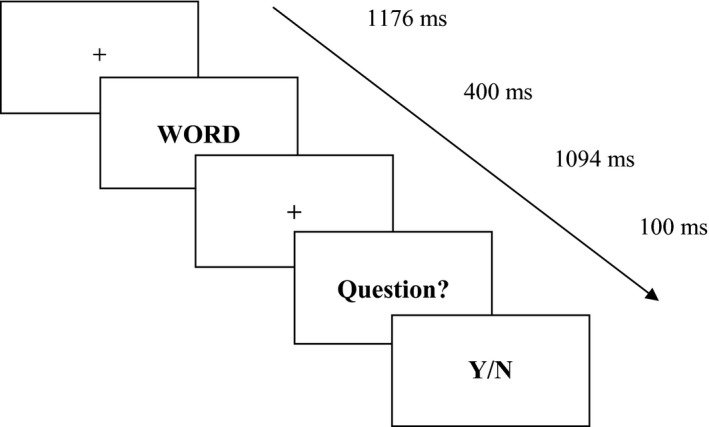
The succession and timing of the main events in the CIT‐like task of critical information concealment. WORD refers to one of the three types of stimuli (familiar, critical, or neutral type); “Question” refers to the question shown to the subjects after each word; Y/N refers to the response stage where subjects had to answer the question (always denying having seen the item they “stole” when the critical word was presented)

Three types of stimuli to be presented during the concealed information task in terms of their status with regard to familiarity, stealing and the required response were specified for the experiment: (1) words which were familiar stimuli from the “thieving” episode, but not “stolen” (two words), (2) the word corresponding to the good actually taken when “thieving” (one critical, or probe word stimulus for each subject), (3) two words referring to items new to the subjects (neutral word stimuli that were not present in the behavioral context of thieving for that subject). After the “thieving” episode, EEG caps were fitted to the subjects, followed by the blocks of sham/rTMS (with targets in DLPFC, Figure 1), followed in turn by recording of ERPs in response to the visual word stimuli in the CIT‐like task stage of the experiment (Figure 2).

Each block of the CIT‐like experimental task was preceded by and associated with offline rTMS which is known as a suitable stimulation method in order to have an inhibitory effect on the cortical areas involved in deception (Hallett, 2007; Karton & Bachmann, 2011; Luber et al., 2009). In one group of subjects (*n *= 9), right DLPFC (rDLPFC) was stimulated either by sham stimulation or real rTMS; in the other group of subjects (*n *= 9), left DLPFC (lDLPFC) was stimulated. The stimulation blocks in each group were given according to AABB/BBAA design. Each subject participated in two sessions that were carried out on different days (i.e., one session per day). Each session belonged to one of the two conditions (sham – A and rTMS – B), with two blocks in each. All rTMS blocks (either sham‐rTMS or real rTMS) were immediately followed by the behavioral CIT‐like task. In each block, the train of 1‐Hz rTMS pulses (360 pulses, 6 min) or sham stimulation pulses (coil held perpendicularly next to the head and a recorded 1‐Hz train of audible TMS coil clicks presented over earphones) were delivered before each behavioral task. Stimulation was targeted either at lDLPFC or at the rDLPFC (see Figure 1). To mask the coil‐generated clicks and reduce differences between real rTMS and sham, music was played through earphones in both conditions. MRI‐assisted NBS (Navigated Brain Stimulation) Nexstim Ltd. (Helsinki, Finland) system with figure‐of‐eight coil was used for stimulation. As intensities close to the motor threshold (MT) may be used as a guide for the stimulus intensity needed for prefrontal TMS (Kähkönen, Wilenius, Komssi, & Ilmoniemi, 2004), the stimulation intensity was set at 80% of the individual MT (measured as a barely noticeable twitch of the thumb). The intensity of stimulation used for different subjects ranged between 29% and 42% of maximal stimulator output.

The offline stimulation protocol (a train of 1‐Hz rTMS delivered *before* the behavioral task and not during it) was used in order to capitalize on what we know from earlier research. The rTMS format is suitable for obtaining an inhibitory effect on the selected cortical area and also for avoiding the nonspecific concurrent effects of TMS when EEG is recorded later during task performance. The offline method also guarantees that subjects perform a task similar to what has been used in CIT and “guilty knowledge” detection studies where psychophysiological responses to critical stimuli are recorded, but concurrent TMS is not used. As the intensity and duration of stimulation is limited (because of overheating of the TMS coil with repeated pulses), we tried to find the best possible solution to generate the effect that will probably last enough. This is necessary in order to obtain sufficient number of behavioral trials for EEG analysis and at the same time have a sufficiently strong effect on the functionality of the target locus in the cortex. There is evidence that DLPFC is reacting already at 40% value of the TMS pulse intensity calibrated against the MT when targets are in the motor cortex (Komssi & Kähkönen, 2006). Less is known about the duration of offline stimulation effects in PFC. According to Robertson, Thĕoret, and Pascual‐Leone (2003) and Thut and Pascual‐Leone (2010) the effect of stimulation in DLFPC diminished after 5–10 (15) minutes from the end of stimulation. Hansenne et al. (2004) maintained that 1‐Hz rTMS produces inhibitory effect only when the duration of the stimulation is about 15 min; according to Eisenegger, Treyer, Fehr, and Knoch (2008) the prefrontal rTMS causes increase in rCBF under the stimulation site, the effect lasting about 9 min. In order to collect enough data for EEG analysis per subject, the need to split the experiment between 2 days appeared to be necessary.

Subjects were seated at the distance of 70 cm from the computer monitor (Eizo FlexScan T550, 1024 × 768 pixels, 85 Hz refresh rate). When delivery of rTMS ended, subjects were engaged in the next experimental step consisting in the CIT‐like task. Foveally located word stimuli belonging to all stimuli types were repeatedly presented on a computer screen in random order. Each stimulus was presented for 400 ms, 18 times per block. Thus, there were 90 stimuli in each block. The words were presented as high‐contrast dark letters on a light background. The luminance of the background was 80 cd/m^2^. Each stimulus was preceded by a screen view with fixation cross in the middle of the screen (1176 ms). Subjects knew that they had to withhold from overt responding until they answered a question which was presented later. (Too fast succession of the stimuli and the questions would have contaminated EEG recordings so that ERPs in response to the word stimuli would have been difficult to analyze.) The word display was followed again by the screen with fixation cross (1094 ms). Thereafter, one of the two questions appeared for 1000 ms: “Was this word written on one of the cards?”, or “Is the card with this word held by you?” (see Figure 2). In the first case, subjects should answer earnestly, in the second case, they should always deny having the card. Responses were typed in by response keys on a standard computer keyboard. After responding, subjects initiated the next trial by pressing the space bar. Subjects completed the task within the time sufficient for rTMS effects to sustain. (Individual times varied between 7.41 and 14.49 min, M = 10.51).

### 2.3 EEG and data analysis

In our study, we analyze and present ERP data by aligning time epochs of interest with presentation of the different types of stimuli and do not set our ERP epochs as aligned with responses to the questions. One reason why the epoch was set to visual stimulus presentation was related to a technical limitation: we could use only one effective TMS/EEG trigger/marker from the experiment‐running computer and we decided to time it with the main test event—the perception of the stimulus that either corresponds to the “stolen” item or not. Recognition of the critical object as a concealment‐related cognitive process appeared to us as the main process to tap by the ERPs. On the other hand, extending the epoch for long enough (so as to include also response time) would increase the amount of ERP epochs with artifacts to the extent that the remaining clean epochs do not allow for statistical tests with any significant results. Thus, our results speak mainly about the cognitive part of processing critical stimuli as reflected in P300 and much less about decisions to deceive or not to deceive. Also, as the task instruction required to always deny that a seen stimulus corresponds to the “stolen” one, the decision aspect of the deception‐related processes cannot be comfortably studied with our design. Moreover, P300 is typically brought about by the stimulus‐to‐be‐processed. Furthermore, when subjects saw the stimuli words, they had not been presented with the questions as yet. As the two types of questions were equiprobable and hard to predict, the procedure is valid for measuring just the ERPs produced in response to the different categories of the perceived stimuli.

For recording, we used the Nexstim eXimia EEG‐system with 60 carbon electrodes cap (Nexstim Ltd.). The impedance at all electrodes was kept below 10 kΩ. The EEG signals were referenced to a reference electrode placed on the forehead. The sampling rate was 1450 Hz. All signals were amplified with a gain of 2000 and filtered with a hardware‐based bandpass filter of 0.1–350 Hz. The vertical electro‐oculogram (VEOG) was recorded via two additional electrodes placed above and below the participants' left eye. All recorded EEG data were analyzed with Fieldtrip (http://fieldtrip.fcdonders.nl; version 14‐12‐2013), an open‐source MATLAB toolbox.

Bioelectrical activity was recorded from 15 electrodes: frontal (electrodes AF1, F1, F5, AF2, F2, F6), parietal (electrodes P3, PO3, P4, PO4), temporal (electrodes TP7, TP8), and central (electrodes C3, C4, CZ). After the initial recording, data were low‐pass filtered (zero phase shift Butterworth filter 30 Hz) and segmented into trials from −200 ms to 1000 ms relative to stimulus onset. The data were manually checked for any artifacts, including eye movements and blinks. All trials contaminated by artifacts were discarded from further analysis. Data were baseline‐corrected with a 100 ms window prior to the stimulus onset. For cleaner ERP traces in figures, data were filtered with a 10‐Hz low‐pass filter instead of the 30 Hz low‐pass filter used for data analysis. On average, the following number of trials were available in the experiment: for the neutral condition: *M* = 111.3, *SD* = 22.9; for the familiar condition: *M* = 110.9, *SD* = 23.3; for the critical condition: *M* = 56.0, *SD* = 12.1. The number of available trials was very similar for TMS and SHAM conditions.

Peak‐to‐peak amplitude was used for the analysis of P300 (as recommended by Soskins et al., 2001). The algorithm first identified the 100 ms long segment using the following constraints: the segment is located in the epoch between 300 and 800 ms after stimulus onset and has the highest positive average amplitude. P300 latency is defined as the midpoint of this segment. Next, the algorithm identified the 100 ms long segment between P300 latency and 1000 ms after stimulus onset, which had the highest negative average amplitude. P300 peak‐to‐peak amplitude is defined as the difference between the highest positive and the highest negative average amplitude. Note that, for this procedure data were first averaged over the electrodes within each electrode group. Note also, that the algorithm was applied on each individual ERP.

### 2.4 Statistical analysis

Statistical analyses were performed with R (version 3.0.3), a freely available and powerful statistical programming language. Repeated‐measures analysis of variance (ANOVA) was used to assess the effects of our experimental conditions. If the sphericity assumption was violated according to Mauchly's test for sphericity, p‐values were corrected with the Greenhouse‐Geisser method. Only the corrected p‐values are reported. As recommended by Bakeman (2005), generalized eta‐squared is used to report effect sizes of our ANOVA results. Planned comparisons and post hoc contrasts were carried out via dependent samples t‐test. Post hoc contrasts were corrected with the Holm–Bonferroni method. Unless indicated otherwise, only the corrected *p*‐values are reported. Cohen's d is reported as an estimate of effect size for the dependent samples t‐tests.

## 3 Results

**Figure 3:**
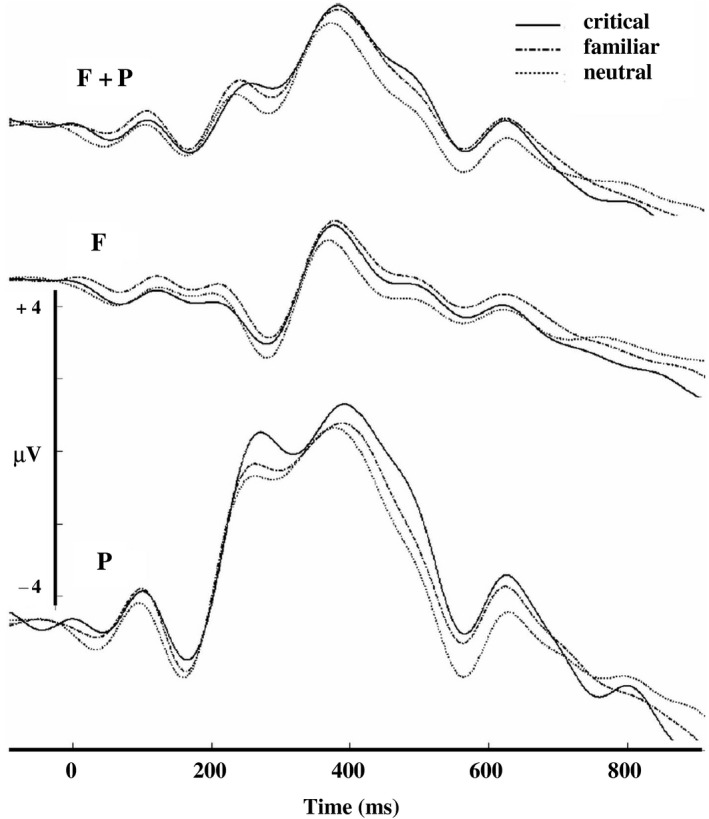
The grand average ERP waveforms per stimulation conditions. (The common voltage scale (μV) is used for all three ERPs. To read the approximate ERP voltage values, the voltage scale must be virtually shifted so that its zero value point would be aligned with the prestimulus baseline level of an ERP)

First, a four‐way repeated‐measures ANOVA with the factors electrode group (frontal and parietal), stimulus type (critical, familiar, neutral) and stimulation type (TMS and SHAM) as within‐subject factors and stimulation side (left and right) as a between‐subjects factor was performed for assessing differences in P300 amplitude. The ERP waveforms per condition can be seen in Figure 3. The main effect of electrode group was significant (*F*_1,16_ = 58, *p *= 1.0e‐06; ηG² = 0.45). This was due to the much lower peak‐to‐peak amplitude of frontal electrodes (*m* = 4.5, *SD* = 1.9) compared to parietal electrodes (*m* = 10.1, *SD* = 3.8). The main effects for stimulation condition and stimulation side were not significant (*F*_1,16_ = 1.1, *p *= .32; ηG² = 0.002 and *F*_1,16_ < 1.0, respectively). There was a near‐significant trend for the main effect of stimulus type (*F*_2,32_  = 3.0, *p *= .07; ηG² = 0.009), but there was a significant interaction between stimulus type and TMS stimulation type (*F*_2,32_ = 3.6, *p *= .04; ηG² = 0.007). All other interactions were not significant (all *F*'s < 1.9, all *p*'s > .19, all ηG² < 0.004).

Planned comparisons were carried out to investigate which conditions differed significantly from each other in terms of the P300 amplitude they elicited. Because no significant interactions between electrode groups, stimulus type and/or stimulation side were found, paired t‐tests were carried out on the average P300 amplitudes over both electrode groups and both stimulation sides. First, we investigated which stimulus types elicit differing P300 amplitudes in response to TMS versus SHAM stimulation. There was a significant difference between the conditions of critical stimuli presented after TMS and after SHAM stimulation (*t*_17_ = −2.3, *p *= .04, *d *= 0.53). The differences in P300 between the conditions of familiar stimuli (after TMS vs. after SHAM, *t*_17_ = −0.5, *p *= .63, *d *= 0.12) and the conditions of neutral stimuli (after TMS vs. after SHAM, *t*_17_ = 1.2, *p *= .25, *d *= 0.28) were not significant.

**Figure 4:**
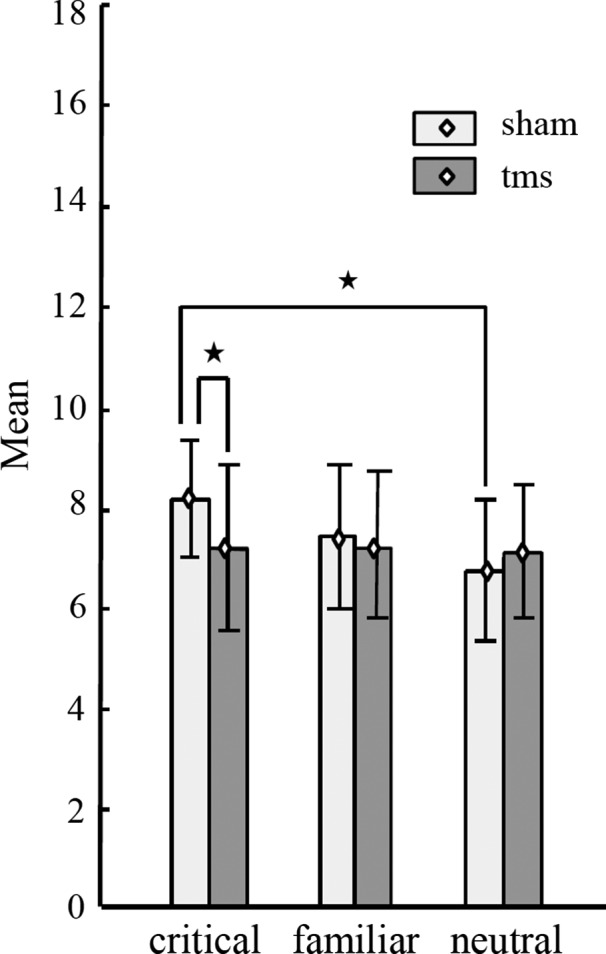
Mean peak‐to‐peak amplitudes (μV) and standard errors for the critical, familiar, and neutral stimulus conditions per stimulation condition (SHAM/TMS). Amplitudes are averaged over frontal and parietal electrode groups (paired t‐test differences, *p < .05)

Second, we investigated if P300 recorded in response to different stimulus types are different within stimulation conditions. However, P300 in the conditions of the three stimulus types did not differ from each other in the TMS condition (critical vs. neutral: *t*_17_ = 0.2, *p *= .84, *d *= 0.05; critical vs. familiar: *t*_17_ = −0.06, *p *= .96, *d *= 0.01; familiar vs. neutral: *t*_17_ = 0.31, *p *= .76, *d *= 0.07), in the SHAM condition, this difference was significant when P300 was compared between the conditions of critical stimuli and neutral stimuli (*t*_17_ = 4.6, *p *= .0002, *d *= 1.1). The comparisons between the conditions of critical and familiar (*t*_17_ = 1.8, *p *= .09, *d *= 0.42) and familiar and neutral stimuli (*t*_17_ = 1.7, *p *= .11, *d *= 0.4) were not significant in the SHAM condition. Figure 4 shows the values of P300 amplitudes and significant differences between our experimental conditions.

Thus, the results show that the P300 response to critical stimuli has higher amplitude if compared with the P300 amplitude in response to neutral stimuli. However, this effect was abolished when DLPFC has been inhibited by rTMS prior to stimulus presentation. Interestingly, the P300 response to critical items exhibits decreased amplitude after rTMS to lDLPFC as well as rDLPFC.

## 4 Discussion

This study aimed at investigating whether noninvasive brain stimulation by offline rTMS has an impact on the relative expression of the best known ERP marker of deception—the augmented P300 to critical stimuli. We distinguished between three categories of stimuli (critical, familiar, and neutral) and expected to find differences in effect between the critical and the neutral stimuli. Using rTMS, functionality of the brain areas rDLPFC and lDLPFC was disrupted. Both these areas (and possibly also sites associated with them) are known to be involved in deception. This intervention was motivated and is substantiated by the results of our earlier published studies (Karton & Bachmann, 2011; Karton, Palu, et al., 2014; Karton, Rinne, et al., 2014) showing that direct or indirect stimulation especially of the right‐hemisphere DLPFC has an effect on deceptive responses. The results of this study showed that amplitude of the P300 in response to the perception of critical items as compared to the perception of familiar and neutral items, was strongly reduced (effectively eliminated). However, we did not find a specific laterality effect that we expected; both right and left DLPFC stimulation diminished the relative P300 amplitude observed in response to critical and neutral stimulus items. As excitability of contralateral homologous (mirror‐symmetric) brain areas is strongly and reliably influenced by ipsilateral TMS (Rogasch & Fitzgerald, 2013), there may be a carry‐over effect so that right and left hemisphere manipulations become equivalent in certain specific conditions. Consequently, it is not straightforward and easy to disentangle left versus right hemisphere effects on deception. This may have happened also in our present study.

In some of our other studies where especially the rDLPFC involvement in deception was found, a different task was used (compared to the CIT type of task used here) (Karton & Bachmann, 2011; Karton, Palu, et al., 2014; Karton, Rinne, et al., 2014). This difference may be a consequence of the different task demands and cognitive processes associated with these tasks. Indeed, as the recent paper from our laboratory showed, when the context of the deceptive behavior was changed so as to increase motivation to lie, left‐hemisphere DLPFC manipulations became more effective in changing the rate of untruthful responses (Karton, Palu, et al., 2014). One more reason why the expected laterality effect of TMS stimulation did not appear this time could be attributed to the fact that frontally targeted TMS is capable of reducing the P300 also in the context of tasks without deception being involved (Torii et al., 2012). Consequently, our effect might be interpreted as an effect on P300 as such, but not specifically related to the *deception‐related* P300. However, as our TMS effect of the deception‐marker reduction was obtained in a typical CIT context and as in the SHAM condition, this marker remained valid, we must conclude that laterality of stimulation of the DLPFC may not be the critical factor when one wants to manipulate the suspect's sensitivity to an ERP‐based CIT test.

It is known that right DLPFC is involved in cognitive control, avoidance, and behavioral inhibition (Cho et al., 2012; Knoch & Fehr, 2007; Ott, Ullsperger, Jocham, Neumann, & Klein, 2011; Shackman, McMenamin, Maxwell, Greischar, & Davidson, 2009; Tassy et al., 2012), while left DLPFC participates in reality monitoring, approach motivation / aggression, strategic behavior, naming, and execution (Berkman & Lieberman, 2010; Fertonani, Rosini, Cotelli, Rossini, & Miniussi, 2010; Hortensius, Schutter, & Harmon‐Jones, 2012; Huffmeijer, Alink, Tops, Bakermans‐Kranenburg, & van IJzendoorn, 2012; Ito et al., 2012; Ott et al., 2011; Steinbeis, Bernhardt, & Singer, 2012). Deception requires considerable cognitive control to inhibit habitual and reality‐corresponding behavior. Thus, it can be expected that right DLPFC stimulation by 1‐Hz rTMS will decrease P300 in response to critical stimuli relative to neutral stimuli compared to the control condition when sham stimulation is used. Yet, why left DLPFC disruption does not counteract this effect remains a puzzle. It is also conceivable that the left versus right dichotomy is related more to the response selection aspect of deceptive behavior, but less to the perceptual aspect of processing critical versus neutral stimuli.

## 5 Conclusions

We saw that in the sham condition, the P300 component exhibited systematic amplitude differences in response to critical items compared to neutral items. As this difference was absent in the rTMS condition when DLPFC was disrupted, we obtained support for the view that DLPFC is involved in CIT‐type deceptive behavior and P300 is a sensitive signature of this. It must be stressed that compared to the use of ERPs as a correlational measure of brain processes related to a specific behavior, TMS provided a means to exert a causal effect on the respective brain systems. This strengthens the arguments in favor of regarding DLPFC and P300 as the substantial factors in CIT‐type deceptive behavior analysis and intervention. In one way or another, this study recommends that P300 amplitude difference is used as the valid EEG signature of deception, and shows that pre‐CIT rTMS as a disruptive type of TMS protocol can decrease the sensitivity of the testee's brain to the test. The obvious agenda for follow‐up studies should be to apply facilitative protocols of TMS instead, in order to see whether P300 sensitivity can be increased.

## Conflicts of Interest

None declared.
